# Transcriptome Analysis of Differentially Expressed Genes Provides Insight into Stolon Formation in *Tulipa edulis*

**DOI:** 10.3389/fpls.2016.00409

**Published:** 2016-03-31

**Authors:** Yuanyuan Miao, Zaibiao Zhu, Qiaosheng Guo, Yunhao Zhu, Xiaohua Yang, Yuan Sun

**Affiliations:** ^1^Institute of Chinese Medicinal Materials, Nanjing Agricultural UniversityNanjing, China; ^2^College of Pharmacy, Henan University of Chinese MedicineZhengzhou, China

**Keywords:** transcriptome sequencing, *Tulipa edulis* (Miq.) Baker, stolon formation, DEGs, gene expression

## Abstract

*Tulipa edulis* (Miq.) Baker is an important medicinal plant with a variety of anti-cancer properties. The stolon is one of the main asexual reproductive organs of *T. edulis* and possesses a unique morphology. To explore the molecular mechanism of stolon formation, we performed an RNA-seq analysis of the transcriptomes of stolons at three developmental stages. In the present study, 15.49 Gb of raw data were generated and assembled into 74,006 unigenes, and a total of 2,811 simple sequence repeats were detected in *T. edulis*. Among the three libraries of stolons at different developmental stages, there were 5,119 differentially expressed genes (DEGs). A functional annotation analysis based on sequence similarity queries of the GO, COG, KEGG databases showed that these DEGs were mainly involved in many physiological and biochemical processes, such as material and energy metabolism, hormone signaling, cell growth, and transcription regulation. In addition, quantitative real-time PCR analysis revealed that the expression patterns of the DEGs were consistent with the transcriptome data, which further supported a role for the DEGs in stolon formation. This study provides novel resources for future genetic and molecular studies in *T. edulis*.

## Introduction

*Tulipa edulis* (Miq.) Baker is a perennial herb in the Liliaceae family. The bulb has been used in traditional Chinese medicine (TCM) under the common name “*Guangcigu*” and is widely used in the treatment of sore throat, scrofula, ulcer and postpartum blood stasis (Chinese Herbalism Editorial Board, 1999). Currently*, Guangcigu* is also an important medicine for the treatment of a variety of tumors, such as throat cancer, lymphoma, and breast cancer (Yeh, 1973; Miao et al., 2015). Currently, the acquisition of *Guangcigu* is mainly dependent on wild resources. Due to the high market demand over the past few decades, there has been a rapid decline in the natural resourses of *T. edulis* (Bing et al., 2008; Ma et al., 2014). Improving the reproductive index of *T. edulis* is an urgent issue for its artificial cultivation.

**FIGURE 1:**
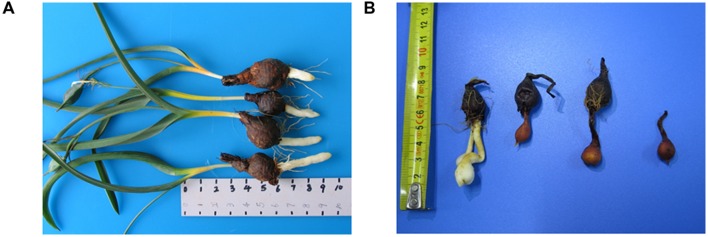
**(A)** *Tulipa edulis* with elongated stolon; **(B)** *T. edulis* stolon developed into a new bulb.

The stolon is one of the main asexual reproductive organs of *T. edulis* and possesses a unique morphology. The stolon is similar to the rhizome in appearance; however, the former has no visible node, internode, or adventitious root. The stolon begins at the stem, bulges outward and extends more than 10 cm at the bottom of the stem. Then, the stolon is pushed deeply in the soil or culture medium and develops a bulblet at the tip. Finally, the stolon shrivels up, and the bulblet, which is surrounded by a thin brown tunic remains (**Figure 1**; Custers et al., 1992; Miao et al., 2015). The stolon plus the bulblet together is called the dropper (Custers et al., 1992). In many studies, the formation of the stolon has been described in micropropagation culture in some *Tulip* spp. plants as a transition to bulblet formation (Custers et al., 1992; Koster, 1993). Nevertheless, there are few studies of the stolon in the tulip under natural conditions. In the tulip, stolon formation was observed in seedlings and small bulbs that grow in wet soil as well as in large bulbs (Le Nard and De Hertogh, 1993). The number of stolons significantly affects the reproductive index. In addition to being a reproductive organ, the stolon of *T. edulis* plays an important role in its adaptation to diverse conditions. Organ-specific gene expression contains rich information on *in vivo* biological processes. Organ differentiation in multi-cellular organisms depends on highly organized molecular events and lends enhanced functionalities to the organisms in their adaptation to natural environments (Guan and Lu, 2013). To date, only a few studies have described the structure of the stolon in similar plants (Custers et al., 1992; Koster, 1993), and research on the morphological characteristics and the development period of *T. edulis* stolon has not yet been performed. The poor understanding of the molecular mechanisms and regulatory networks limits the research on *T. edulis* stolon formation and further hampers the use of reliable technologies to improve the reproductive index of *T. edulis*. Therefore, it is necessary to explore gene networks and biological processes to promote the further study of stolon growth and to meet the strong market demand.

One of the major challenges for *T. eduli*s developmental study is that it does not have genomic sequences and gene annotation, which cannot provide valuable information for the investigation of molecular mechanisms in *T. edulis*. Transcriptome sequencing (RNA-seq) is a feasible and economic sequencing technology with which to discover new genes and provide a platform for understanding differential gene expression at the genomic and transcriptional level. RNA-seq has been widely applied in plant biology and has shown great potential for expanding sequence databases of not only model species but also non-model organisms, such as celery (Li M.Y. et al., 2014) and Ma bamboo (Liu et al., 2012). The data obtained from RNA-seq can be used to interpret the functional elements of the genome and reveal the molecular constituents of cells and tissues (Wang et al., 2009; Wei et al., 2011). Zhang R. et al. (2013) used transcriptome sequencing and bioinformatics analysis to analyze potato-specific miRNAs and corresponding target genes in the potato tuber formed from underground stolons. Gong et al. (2015) examinated differentially expressed genes (DEGs) at potato tuber bulking stages by transcriptome sequencing of stolon materials. In addition to stolon, transcriptome sequencing was applied to other underground organs originated from underground stems, including tuberous root, rhizome and bulblet formation (Cheng et al., 2013; Firon et al., 2013; Li X.Y. et al., 2014). RNA-seq has also been successfully used in research on certain organs, such as fiber development (Naoumkina et al., 2013; Nigam et al., 2014), and rhizome and aerial-shoot development (Zhang T. et al., 2013). More quantitative and detailed information about gene expression can now be collected for each organ or organ developmental stages than previously available (Liu et al., 2012; Guan and Lu, 2013).

In the present study, we determined the transcriptomes of *T. edulis* stolons at three developmental stages using a sequencing platform for the purpose of investigating the DEGs associated with stolon formation. A total of 74,006 unigenes were identified in *T. edulis.* We compared the gene transcription abundances of different developmental stages and identified numerous DEGs at different developmental stages of stolon formation. The functional annotation and classification of the DEGs were beneficial for a better understanding of their biological significance. Furthermore, quantitative real-time PCR (qRT-PCR) was performed to analyze the expression patterns of DEGs involved in stolon formation. To the best of our knowledge, this is the first report of the characterization of the complete transcriptome of *T. edulis*. We believe that the systematic analysis of transcriptomes may contribute to our understanding of the molecular mechanisms underlying stolon formation in *T. edulis*.

## Materials and Methods

### Plant Material Preparation

The experiment was performed under natural day/night conditions in the greenhouse of the Institute of Chinese Medicinal Materials at Nanjing Agricultural University, Nanjing, P.R. China. *T. edulis* bulbs were obtained from Funan county, Anhui province in September 2013. Disease-free bulbs of uniform size (∼1.0–1.5 g) were selected for planting in polyethylene plastic disks filled with nutrient soil. The field capacity (FC) of the nutrient soil was 26.56%, and the water content of the nutrient soil was gravimetrically adjusted every other day to 80% FC. The disks were arranged following a completely randomized block design. Stolon formation was divided into the following three stages: (1) T1, the initial period of stolon formation, 41 days after planting (DAP); (2) T2, the middle period of stolon formation, 118 DAP; and (3) T3, the later period of stolon formation, 133 DAP. Stolons from these three stages were collected, immediately frozen in liquid nitrogen and then stored at -80°C.

### Anatomical Structure Analysis

The study of the anatomical structure of stolons was carried out using paraffin sections based on the methods of Ye (2012). Fresh samples were quickly placed into formalin-acetic acid-alcohol (FAA) fixative for 24 h and placed under a low vacuum to ensure the penetration of the fixative. Then, the samples were dehydrated by a graded ethanol series and embedded into paraffin blocks. Samples were cut into 8–10 μm with a rotary microtome, deparaffinized, stained with safranin O and astra blue and mounted with synthetic resin under a coverslip. The slices were observed and then photographed using an Olympus BX-51 microscope (Olympus, Tokyo, Japan).

### RNA Isolation and Transcriptome Sequencing

The total RNA for stolons of three stages was extracted using the RNAsimple total RNA kit (Tiangen, Beijing, China) according to the manufacturer’s instructions. RNA quantity and quality were determined by gel electrophoresis and spectrophotometer analysis (Nanodrop ND 1000 spectrophotometer, Nanodrop Technologies, Rockland, ME, USA). The mRNA in the samples was concentrated using magnetic oligo-dT beads and then cleaved into fragments, and served as a template for the synthesis of the first-strand cDNA using random hexamers and reverse transcriptase. The second-strand cDNA was synthesized using dNTPs, RNase H, and DNA polymerase I. A suitable length of cDNA fragments was selected by a garose gel electrophoresis and amplified by PCR to construct the final cDNA libraries for sequencing on the Illumina HiSeq 2500 platform (Biomarker Technologies Co., Ltd, Beijing, China). The transcriptome sequencing data have been submitted to NCBI under the accession number SRR2177455.

### *De Novo* Assembly and Analysis

Raw reads from three samples (24,156,071 raw reads for T1, 26,494,762 raw reads for T2 and 26,021,724 raw reads for T3) were collected. A Perl script was written to removing low quantity reads, that is, reads with more than 20% of bases with a Q-value ≤20 or the content of ambiguous sequence “N” exceeding 5%. Then the remaining high-quality reads were assembled into contigs using the Trinity software (Grabherr et al., 2011) with the parameters set at a similarity of 90%. Subsequently, the contigs were assembled into transcripts by performing pair-end joining and clustered to unigenes. Finally, the randomness test was performed to check the mapped reads positions.

### Functional Annotation

Functional annotation was performed by sequence similarity search with public databases. All assembled unigenes were compared with NCBI non-redundant protein database (Nr) (Deng et al., 2006), SWISS-PROT (Apweiler et al., 2004), Gene Ontology (GO) (Ashburner et al., 2000), Clusters of Orthologous Groups (COG) (Tatusov et al., 2000) and the Kyoto Encyclopedia of Genes and Genomes (KEGG) databases (Kanehisa et al., 2004). Blast2GO was used to obtain GO functional categories (Conesa et al., 2005), and WEGO software was then used to illustrate the distribution of the gene functions (Ye et al., 2006).

### Simple Sequence Repeats (SSR) Screening

Potential SSR markers were detected among all assembled unigenes using the MIcroSAtellite (MISA) tool (http://pgrc.ipk-gatersleben.de/misa/) (Thiel et al., 2003). SSRs with motifs ranging one to six nucleotides were analyzed. The parameters of repeat units were set for mono-, di-, tri-, tetra-, penta-, and hexa-nucleotide motifs with a minimum of 10, 6, 5, 5, 5, and 5 repeats, respectively.

### Transcript Abundance Analysis and DEGs Selection

Transcript abundance of all unigenes in stolon in different stages of *T. edulis* were estimated by calculating the read density as ‘reads per kilobase of exon model per million mapped reads’ (RPKM) (Mortazavi et al., 2008). The DEGs were identified among three libraries using EBSeq software (Leng et al., 2013). A threshold for false discovery rate <0.01 and a fold-change ≥2 were used to determine significant expression changes.

### Determination of Carbohydrate Contents

Approximately 0.5 g of frozen samples were homogenized with 4 mL 80% ethanol, extracted at 80°C for 30 min, and centrifuged at 8,000 rpm for 20 min. The supernatant decolorized by activated carbon was used for the measurement of soluble sugars including sucrose, reducing sugar and fructose. The precipitates were successively suspended in 9.2-M and 4.6-M perchloric acid to extract the starch after removing the ethanol soluble sugar residues. An anthrone reaction was used for the determination of the total soluble sugar and starch concentrations. The content of reducing sugar was determined using 3,5-dinitrosalicylic acid, and the sucrose and fructose concentrations were measured through hydrolysis and resorcinol reactions.

### qRT-PCR Analysis

Quantitative real-time PCR was performed using the ABI 7300 Real-Time PCR System (Applied Biosystems, Foster City, CA, USA) with the fluorescent dye SYBR Green (TaKaRa, Dalian, China). The gene-specific primers were designed using Primer Premier 5, and the primer sequences are listed in **Supplementary Table S1**. The PCR cycling conditions were as follows: 95°C for 30 s, 40 cycles of 95°C for 5 s and 58°C for 30 s. Each reaction was repeated three times, and *actin-1* was used as an internal control. The relative gene expression levels were calculated with the 2^-ΔΔCT^ method (Pfaffl, 2001).

## Results

### Developing Characteristics of Stolon in *T. edulis*

**FIGURE 2:**
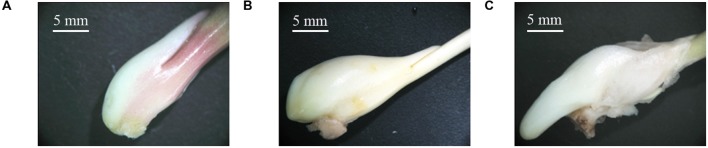
**Morphological changes in the stolon formation of *T. edulis.*** **(A)** *T. edulis* stolon formation at the initial stage; **(B)** *T. edulis* stolon formation at the middle stage; **(C)** *T. edulis* stolon formation at the later stage. Scale: 5 mm.

The process of stolon formation can be divided into three stages: stage T1 (the initial period of stolon formation, 41 DAP, **Figure 2A**), stage T2 (the middle period of stolon formation, 118 DAP, **Figure 2B**), and stage T3 (the later period of stolon formation, 133 DAP, **Figure 2C**). Using a stereo microscope, we observed that in stage T1, the location of stolon formation was in close proximity to the side of the stem base plate bulb, and the surface of early stem base was smooth (**Figure 2A**). In stage T2, the stem base protruded outward and had a significant increase in the volume (**Figure 2B**). In stage T3, the stolon continued to grow, showing some special characteristics, including a cylindrical shape, a milky white color, a lateral smooth texture, no visible node and internode, and no adventitious root (**Figure 2C**).

**FIGURE 3:**
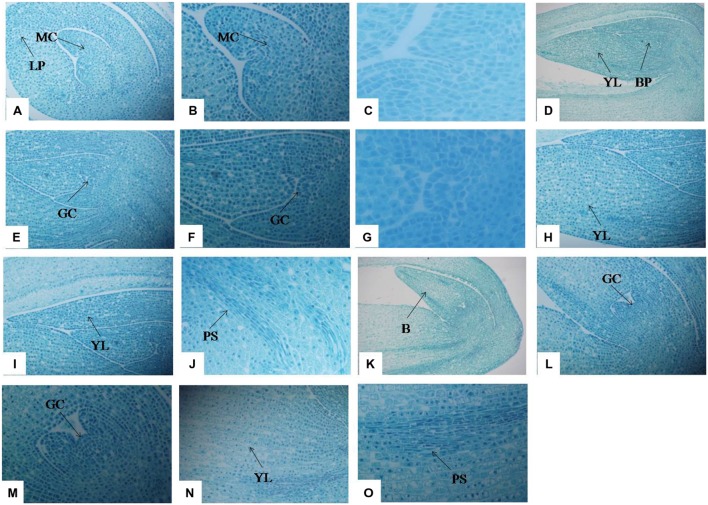
**Anatomical changes in stolon formation of *T. edulis*.** Abbreviations: MC, meristematic cell; LP, leaf primordium; YL, young leaf; BP, bud primordium; GC, growth cone; PS, procambial strand; B, bud. **(A)** Meristematic cell and leaf primordium waiting for cell division in the initial period of stolon formation (10×). **(B)** Meristematic cell waiting for cell division in the initial period of stolon formation (20×). **(C)** Meristematic cell waiting for cell division in the initial period of stolon formation (40×). **(D)** Young leaf and bud primordium in the middle period of stolon formation (4×). **(E)** Growth cone in the middle period of stolon formation (10×). **(F)** Growth cone in the middle period of stolon formation (20×). **(G)** Growth cone in the middle period of stolon formation (40×). **(H,I)** Young leaf in the middle period of stolon formation (10×). **(J)** Procambial strand in the middle period of stolon formation (20×). **(K)** Bud in the later period of stolon formation (10×). **(L)** Growth cone in the later period of stolon formation (10×). **(M)** Growth cone in the later period of stolon formation (20×). **(N)** Young leaf in the later period of stolon formation (10×). **(O)** Procambial strand in the later period of stolon formation (20×).

Optical microscopy revealed the anatomical structure of stolons at different development stages. In T1 (**Figures 3A–C**), a large number of meristematic cells appeared, preparing for the cell division of stolon formation. The cells surrounding both sides of the leaf primordium are also waiting for cell division. In T2, the cells proceed to periclinal division and anticlinal division processes and differentiate into the growth cone (**Figures 3D–G**). The leaf primordium cells that surrounded the bud primordium also appeared in a series of division, and the number of cells in the left young leaf increased significantly compared with the T1 stage (**Figure 3H**). On the right side, along with the increase in the number of young leaf cells, elongation growth occurred and cells formed young leaves (**Figure 3I**). With the development of the growth cone, the procambial strand was observed (**Figure 3J**). In T3, bud primordia cells continued to divide, resulting in a substantial increase in the number of cells, and formed a large bud (**Figure 3K**); within the bud there was a growth cone (**Figures 3L,M**). Growth cone continued to differentiate and formed the bud primordium or stem primordium (**Figure 3M**). Cells were arranged slightly loosely in the young leaf on the left side of the bud, and the procambial strand structure became more developed than at the T2 stage (**Figures 3N,O**). At the T3 stage, stolon elongation typically occurred.

### Transcriptome Sequencing, *De Novo* Assembly, and Functional Annotation

Three cDNA libraries of *T. edulis* were constructed for transcriptome sequencing to reveal the transcriptome differences among three stolon formation stages. In this study, 4.88 Gb, 5.35 Gb, and 5.26 Gb of sequence data were generated from stolons at T1, T2, and T3, respectively. After data filtering, a total of 19,376,548 high quality reads were obtained for T1, 20,554,166 for T2, and 20,293,471 for T3. The high-quality reads for stolons at the three stages were merged to generate the complete *T. edulis* transcriptome data. Using Trinity software, the reads were assembled into 5,115,834 contigs with a mean length of 57 bp and an N50 length of 50 bp. By performing pair-end sequencing, contigs were further assembled into 151,864 transcripts and then clustered into 74,006 unigenes. All the reads were mapped to the unigenes to check the sequencing randomness, and result showed that the sequencing randomness of each sample is well (**Supplementary Figure S1**). These unigenes had lengths in the range of 201–12,340 bp, an average length of 659 bp and an N50 length of 1,005 bp. The unigenes that had a sequence length ranging from 200 bp to 2,000 bp accounted for 94.67% of the unigenes. Up to 13,236 unigenes (17.89%) were greater than 1,000 bp. An overview of the assembled contigs, transcripts, and unigenes are presented in **Table 1**. These results also demonstrated the effectiveness of Illumina pyrosequencing in rapidly capturing a large portion of the transcriptome.

**Table 1:** Summary of the Illumina transcriptome assembly for *T. edulis*.

Length (bp)	Contig		Transcript		Unigene	
	Number	Percentage	Number	Percentage	Number	Percentage
200–300	5,058,562	98.88%	36,775	24.22%	25,519	34.48%
300–500	26,214	0.51%	35,932	23.66%	20,233	27.34%
500–1000	17,836	0.35%	39,689	26.13%	15,018	20.29%
1000–2000	9,499	0.19%	28,344	18.66%	9,294	12.56%
>2000	3,723	0.07%	11,124	7.32%	3,942	5.33%
Total	5,115,834		151,864		74,006	
Total length	290,807,176		122,338,764		48,743,720	
N50 length	50		1,224		1,005	
Mean length	56.84		805.58		658.64	


All of the unigenes were searched against five public databases for functional annotation. The overall functional annotation is described in **Table 2**. Up to 28,427 and 22,460 unigenes had a sequence similarity to known genes when blasting to the Nr database and SWISS-PROT, respectively, with an *E*-value threshold of 1e^-5^. A total of 18,635 unigenes were annotated by GO analysis and classified into three GO categories and 56 functional groups (**Supplementary Figure S2**). Biological process accounted for the largest proportion in GO annotation, followed by cellular component and molecular function. A total of 8,195 and 5,671 unigenes were further annotated by the COG and KEGG databases, respectively. These annotations provide useful information for molecular studies of *T. edulis*.

**Table 2:** Functional annotation of *T. edulis* unigenes by sequence similarity search.

Annotated	Annotated	300 ≤ length	Length ≥
databases	number	< 1000 (bp)	1000 (bp)
Nr	28,427	11,902	11,982
SWISS-PROT	22,460	8,664	10,827
COG	8,195	2,399	5,122
GO	18,635	6,903	9,052
KEGG	5,671	1,891	3,038
Total annotated	28,665	12,044	11,997


### SSR Screening and Analysis

**FIGURE 4:**
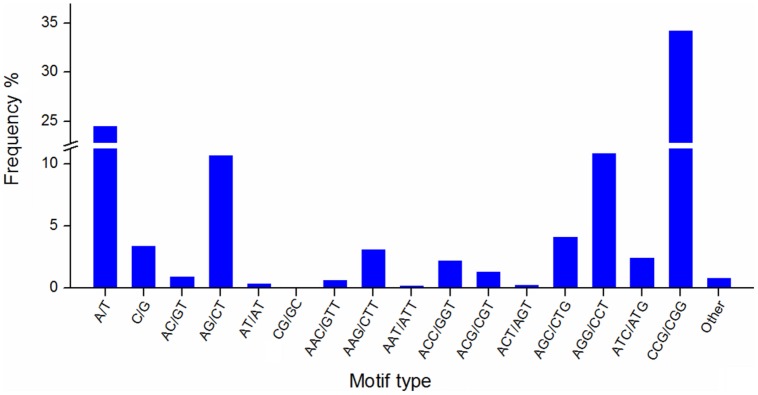
**The frequency of SSR motif types in *T. edulis***.

Simple sequence repeats are widely used in genetic research, linkage map construction, and breeding study. To explore SSRs in *T. edulis*, 13,236 unigenes that had a length more than 1,000 bp were screened using MISA software. A total of 2,811 SSRs were identified in 2,484 unigenes, among which 280 unigenes had more than one SSR. The length of different SSR repeat types varied greatly (**Table 3**). The tri-nucleotide repeat type accounted for more than half (59.34%) of the total SSRs, followed by mono-nucleotide (27.89%) and di-nucleotide (11.95%). The remaining types (tetra-, penta-, hexa-) had a frequency of less than 1%. The frequencies of SSR motif types are shown in **Figure 4**. In mono-nucleotide repeats, the A/T type was the abundant type and accounted for 24.51%. The most frequent type of di-nucleotide repeats, AG/CT, accounted for the vast majority of the proportion and far more than the other three types. Among all motif types, CCG/CGG repeats represented the largest proportion (34.26%) of all motif types. Additionally, another tri-nucleotide motif, AGG/CCT, accounted for 10.85% of all SSRs. The formation of SSR repeat types with a higher G + C content showed an observable base preference.

**Table 3:** Number of different SSR repeat types in the *T. edulis* transcriptome.

Repeat type							Repeat numbers	Total	%
	5	6	7	8	9	10	11	12	13	14	15	>15		
Mono-nucleotide	0	0	0	0	0	378	170	94	41	29	35	37	784	27.89
Di-nucleotide	0	115	79	53	34	31	24	0	0	0	0	0	336	11.95
Tri-nucleotide	1,049	444	163	11	0	0	0	1	0	0	0	0	1,668	59.34
Tetra-nucleotide	7	3	1	0	0	0	0	0	0	0	0	0	11	0.39
Penta-nucleotide	0	1	0	0	0	0	0	0	0	0	0	0	1	0.04
Hexa-nucleotide	3	5	2	1	0	0	0	0	0	0	0	0	11	0.39
Total	1,059	568	245	65	34	409	194	95	41	29	35	37	2,811	100
%	37.67	20.21	8.72	2.31	1.21	14.55	6.90	3.38	1.46	1.03	1.25	1.32	100	


### Identification and Selection of DEGs

**FIGURE 5:**
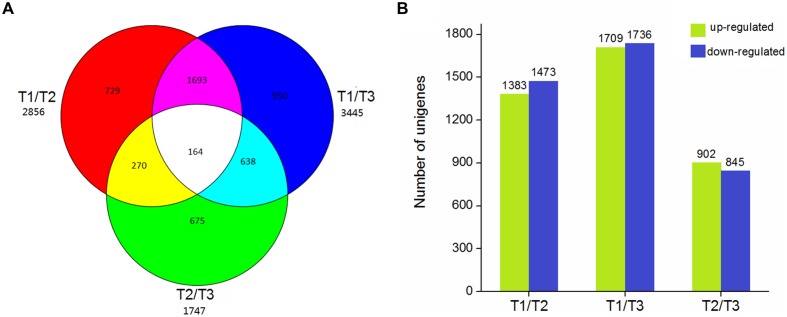
**The DEGs in different comparisons (control/experiment: T1/T2, T1/T3, and T2/T3) during *T. edulis* stolon formation.** T1, T2, and T3 indicate the initial, middle, and later periods of stolon formation, respectively. **(A)** Venn diagram representing the unique and common DEGs among different comparisons. **(B)** The expression patterns of DEGs among different comparisons.

**FIGURE 6:**
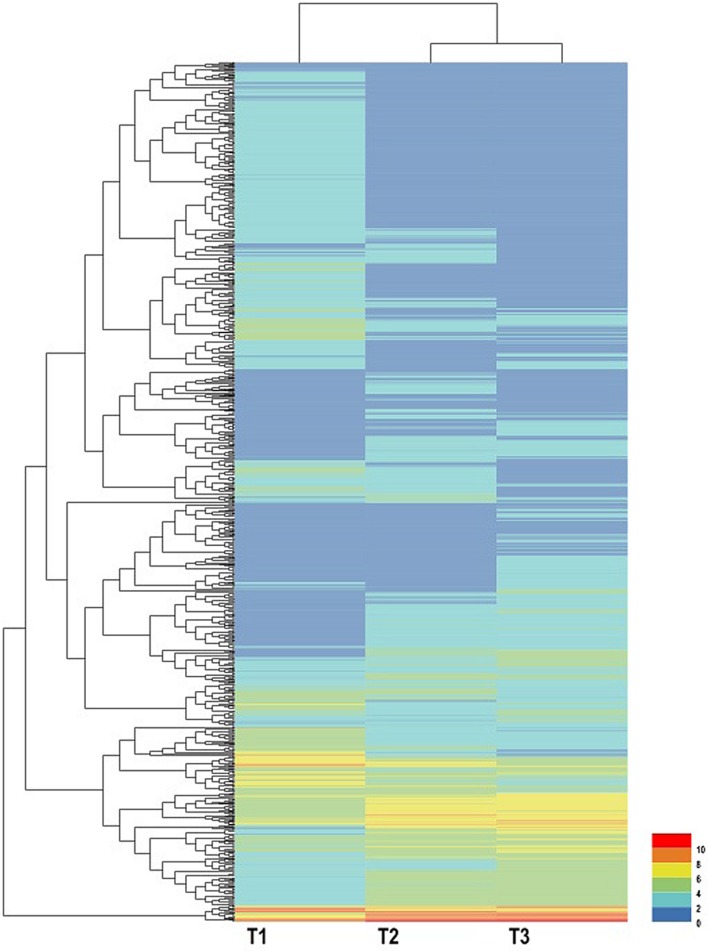
**Expression profiles and cluster analysis of DEGs at different developmental stages of *T. edulis* stolon formation.** T1, T2, and T3 indicate the initial, middle, and later periods of stolon formation, respectively.

Using EBSeq software, 5,119 DEGs were detected in at least one comparison sample (control/experiment: T1/T2, T1/T3, and T2/T3). A Venn diagram was constructed to represent the numbers of DEGs in two or more comparisons (**Figure 5A**). Among the three comparisons, T2/T3 contained the smallest number of DEGs (1,747), whereas 2,856 and 3,445 DEGs were detected in T1/T2 and T1/T3, respectively. Of these, 164 DEGs was specifically detected in all comparisons. This result indicated that gene differences in the initial stage of development were the greatest. Gene transcript abundance was further analyzed as shown in **Figure 5B**. Compared with T1, 1,383 and 1,709 genes were up-regulated, and 1,473 and 1,736 genes were down-regulated in T2 and T3, respectively. While in T2/T3, 902 genes were up-regulated and 845 genes were down-regulated. Transcript abundances of all unigenes in stolon in different stages of *T. edulis* were measured by RPKM (Mortazavi et al., 2008). The RPKM values were first evaluated to check the distributions in three samples (**Supplementary Figure S3**). The transcript abundances of all unigenes were clustered using hierarchical cluster analysis (**Figure 6**). Based on the expression analysis, we also found that T1 had a large difference compared to T2 and T3, while a similar pattern was observed between T2 and T3.

### Gene Annotation and the Functional Classification of DEGs

**FIGURE 7:**
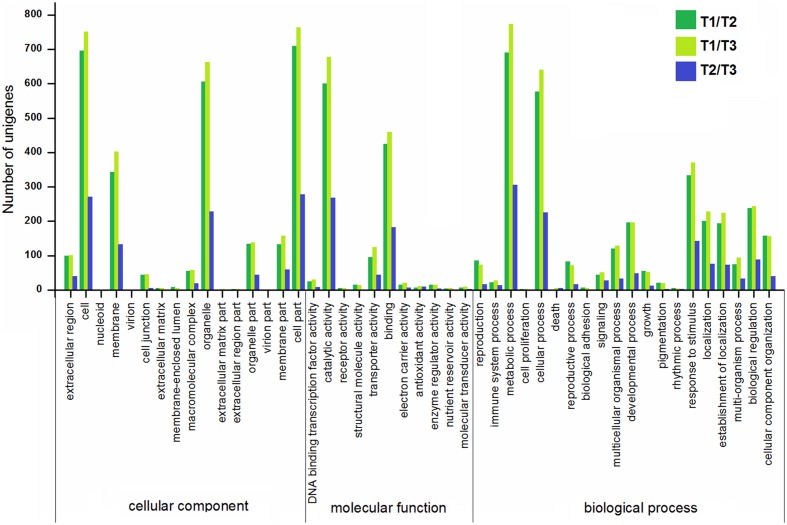
**Gene ontology (GO) enrichment analysis of DEGs among different comparisons (control/experiment: T1/T2, T1/T3, and T2/T3) during *T. edulis* stolon formation.** T1, T2, and T3 indicate the initial, middle and later periods of stolon formation, respectively.

To gain more insights into the DEGs, the GO, COG, KEGG databases were used to annotate the functions of the DEGs. GO enrichment of the DEGs in stolon formation was analyzed. As illustrated in **Figure 7**, the GO annotations for three comparisons were enriched for some particular GO terms, and the numbers of annotated DEGs in T1/T2 and T1/T3 were greater than those in T2/T3. The most DEGs in each comparison are all assigned to a biological process, with the most frequent GO terms being “metabolic process,” “cellular process,” and “response to stimulus.” These genes were predicted to be relevant to stolon formation in *T. edulis*. According to the cellular component GO category, the DEGs were enriched for “cell,” “cell part,” “organelle,” and “membrane” during stolon formation, suggesting that a large number of genes may be involved in development, especially in the initial developmental stage. For the molecular function GO category, DEGs involved in “catalytic activity” and “binding” were highly represented.

**FIGURE 8:**
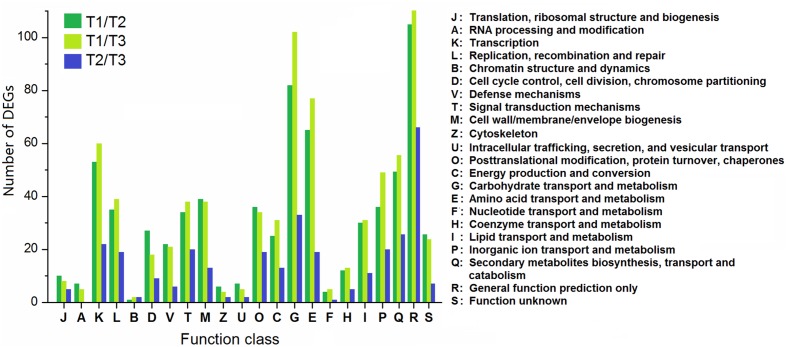
**Clusters of orthologous groups classification of DEGs among different comparisons (control/experiment: T1/T2, T1/T3, and T2/T3) during *T. edulis* stolon formation.** T1, T2, and T3 indicate the initial, middle and later periods of stolon formation, respectively.

According to the COG database, the DEGs were functionally clustered into 22 classifications (**Figure 8**). The first three classifications in T1/T2 and T1/T3 comparisons were “general function prediction only,” “carbohydrate transport and metabolism,” and “amino acid transport and metabolism,” while “general function prediction only,” “carbohydrate transport and metabolism,” and “secondary metabolites biosynthesis, transport and catabolism” were the groups with the most DEGs in T2/T3 comparison. Moreover, the proportions of DEGs in T2/T3 comparison in “signal transduction mechanisms,” “posttranslational modification, protein turnover, chaperones,” “secondary metabolites biosynthesis, transport and catabolism” were higher than T1/T2 and T1/T3 comparisons, suggesting that these genes are important in later developmental stage.

The KEGG database was used to further understand the biological functions and pathways of DEGs. By mapping the enzyme commission numbers to the pathways, 206, 230, and 82 DEGs were assigned to 87, 88, and 58 pathways in T1/T2, T1/T3, and T2/T3 comparisons, respectively. The top 20 KEGG pathways in three comparisons are represented in **Table 4**. The results show that “starch and sucrose metabolism,” “galactose metabolism,” and “plant hormone signal transduction” had larger proportions of DEGs in both T1/T2 and T1/T3 comparisons. However, it was strange that these three pathways accounted for a small part in T2/T3. Notably, “plant hormone signal transduction” was not detected in T2/T3. It is inferred that these three pathways of DEGs are important in early developmental stage of stolon formation. The “glycolysis/gluconeogenesis” term had the maximum DEGs in T2/T3, followed by “phenylpropanoid biosynthesis” and “starch and sucrose metabolism”. However, “glycolysis/gluconeogenesis” was not detected in T1/T2 and T1/T3, suggesting that it played crucial role in later developmental stage. Moreover, four other pathways (“endocytosis,” “ether lipid metabolism,” “tyrosine metabolism,” and “glycerophospholipid metabolism”) also only appeared in T2/T3, implying that some special enzymes play key roles during this particular period. Compared with the percent of unigenes in each pathway, the DEGs had higher proportions, which further illustrated that DEGs might be involved in stolon formation in *T. edulis*.

**Table 4:** Enriched KEGG pathway analysis of DEGs among different comparisons (control/experiment: T1/T2, T1/T3, and T2/T3) during *T. edulis* stolon formation.

KEGG pathway	T1/T2		T1/T3		T2/T3		All unigenes	
	DEGs	Percent	DEGs	Percent	DEGs	Percent	Number	Percent
	Number	(%)	Number	(%)	Number	(%)	Number	(%)
1. Starch and sucrose metabolism	27	13.11	28	12.17	7	8.54	154	4.02
2. Glycolysis/Gluconeogenesis	0	0.00	0	0.00	11	13.41	139	3.62
3. Galactose metabolism	17	8.25	17	7.39	3	3.66	52	1.36
4. Plant hormone signal transduction	15	7.28	18	7.83	0	0.00	160	4.17
5. Phenylpropanoid biosynthesis	11	5.34	11	4.78	8	9.76	74	1.93
6. Amino sugar and nucleotide sugar metabolism	12	5.83	13	5.65	0	0.00	117	3.05
7. Pentose and glucuronate interconversions	10	4.85	12	5.22	6	7.32	53	1.38
8. Phenylalanine metabolism	9	4.37	11	4.78	6	7.32	76	1.98
9. Endocytosis	0	0.00	0	0.00	6	7.32	150	3.91
10. Ether lipid metabolism	0	0.00	0	0.00	4	4.88	62	1.62
11. Tyrosine metabolism	0	0.00	0	0.00	4	4.88	33	0.86
12. Glycerophospholipid metabolism	0	0.00	0	0.00	4	4.88	110	2.87
13. Flavonoid biosynthesis	8	3.88	9	3.91	3	3.66	22	0.57
14. Nitrogen metabolism	8	3.88	9	3.91	3	3.66	40	1.04
15. Glycine, serine and threonine metabolism	8	3.88	9	3.91	0	0.00	46	1.20
16. Terpenoid backbone biosynthesis	7	3.40	0	0.00	3	3.66	47	1.23
17. Ascorbate and aldarate metabolism	6	2.91	6	2.61	0	0.00	39	1.02
18. Alanine, aspartate and glutamate metabolism	6	2.91	6	2.61	0	0.00	48	1.25
19. Carotenoid biosynthesis	5	2.43	0	0.00	2	2.44	38	0.99
20. Glycosaminoglycan degradation	3	1.46	4	1.74	1	1.22	7	0.18


*T1, T2, and T3 indicate the initial, middle and later periods of stolon formation, respectively. Corrected p-value ≤0.05. The top 20 enriched pathways are represented.*

### Dynamic Changes in Starch and Sucrose Concentrations

**FIGURE 9:**
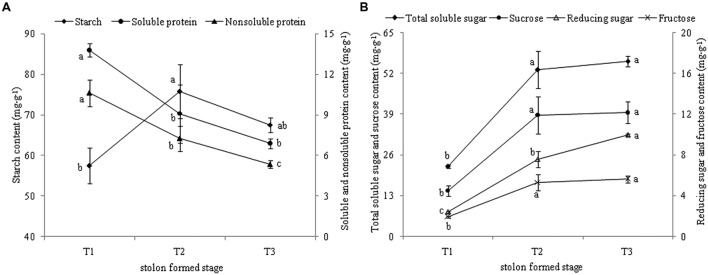
**(A) Dynamic changes of starch and protein content during *T. edulis* stolon formation. **(B)** Dynamic changes of soluble sugars content during **T. edulis** stolon formation.** T1, T2, and T3 indicate the initial, middle and later periods of stolon formation, respectively. Different lowercase letters indicate significant differences (*p* < 0.05) between the three development stages.

KEGG annotation revealed that most DEGs were related to “starch and sucrose metabolism.” The levels of starch and sucrose were analyzed in different stages during stolon formation. The content of starch increased highly from T1 to T2 and reached a maximum in T2, then decreased slightly in T3 (**Figure 9A**). The dynamic changes in soluble protein and non-soluble protein followed a similar trend, as the content decreased faster with stolon formation. The levels of different types of sugars in stolons were measured during the developmental stages (**Figure 9B**). Compared with T1, the concentrations of four types of sugars were significantly up-regulated more than twofold in T2. From T2 to T3, total soluble sugar, sucrose, and fructose had no significant change, but levels also remained high in T3, whereas the content of reducing sugar increased and peaked in T3.

### Expression Analysis of DEGs involved in Stolon Formation

**FIGURE 10:**
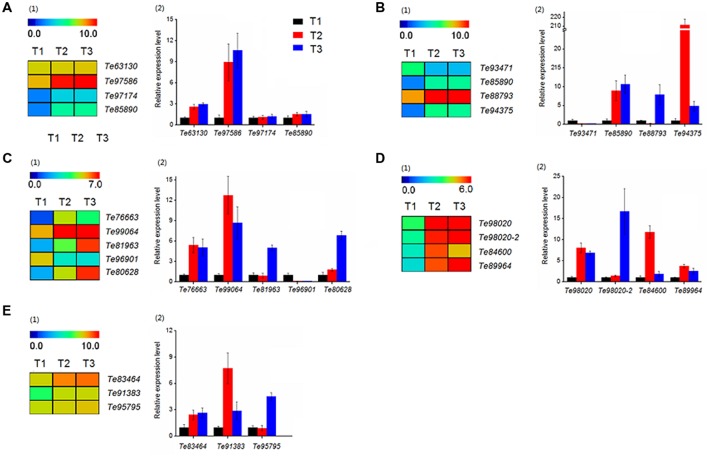
**Heatmap clustering and qRT-PCR analysis of DEGs in *T. edulis* stolon formation.** T1, T2 and T3 indicate the initial, middle and later periods of stolon formation, respectively. **(A)** The DEGs related to cell growth. (1) Transcript abundances of DEGs. (2) Relative expression levels of DEGs. **(B)** The DEGs related to hormone signal. **(C)** The DEGs related to sugar metabolism. **(D)** The DEGs related to sugar synthesis. **(E)** The DEGs related to amino acid metabolism. RPKM values were log_2_-based. The qRT-PCR data are presented as the mean ± SD of three biological and technical replicates.

The results of function annotation suggested that a large number of DEGs associated with stolon formation. In this study, we selected genes for qRT-PCR validation. These genes were predicted to be related to cell growth (*Te63130*, *Te97586*, *Te97174*, *Te85890*), hormone signaling (*Te93471*, *Te85890*, *Te88793*, *Te94375*), sugar metabolism (*Te76663*, *Te99064*, *Te81963*, *Te96901*, *Te80628*), sugar synthesis (*Te98020*, *Te98020-2*, *Te84600*, *Te89964*), and amino acid metabolism (*Te83464*, *Te91383*, *Te95795*). The gene annotations of these DEGs are represented in **Supplementary Table S2**. At the same time, the transcript abundances of these DEGs are calculated with log_2_ ‘relative RPKM.’ According to the results (**Figure 10**), the qRT-PCR analysis and transcriptome data of most genes were consistent.

Among cell growth-related genes (**Figure 10A**), *Te63130* and *Te97586* showed a higher transcript abundance, but the differences in the three stages were not particularly obvious. While for the other two genes, *Te97174* and *Te85890*, the expression levels in T2 and T3 were much higher than the expression in T1, and the differences between T2 and T3 were not obvious.

The expression patterns of four hormone-related genes showed a significant difference at three developmental stages (**Figure 10B**). qRT-PCR showed that compared to T1, the expression level of *Te93471* was down-regulated approximately 5-fold in T2, while in T3, the expression decreased at least 20-fold. However, the remaining three genes showed a significant increase during the stolon formation process.

Genes related to the synthesis and metabolisms of sugar were also evaluated. In sugar metabolism, three genes (*Te99064*, *Te76663*, *Te96901*) showed significant expression changes at the initial stage, while the expression of two others (*Te81963*, *Te80628*) was highly variable (**Figure 10C**). All of the sugar synthesis genes showed very high expression at all three stages (**Figure 10D1**), and qRT-PCR verified this result (**Figure 10D2**).

Regarding amino acid metabolism, *Te83464* and *Te91383* increased significantly from T1 to T2 and maintained higher expression levels in the later stages (**Figure 10E**). Compared with T1, *Te95795* showed no significant change in T2 but was up-regulated more than fivefold in T3.

## Discussion

The stolon is one of the important organs of many plants. In some plants such as strawberry, spider plant, and *T. edulis*, stolon breeding is the main method of reproduction. To clarify the formation of the stolon is not only the important content of plant developmental biology research, but is also a guarantee for improving the quality of plants. In recent years, research on the mechanism of stolon formation has made important progress. Some linkage markers of important traits were developed, and a series of related genes were identified (Eviatar-Ribak et al., 2013; Mabhaudhi and Modi, 2013; Pasare et al., 2013). Starch and sucrose are essential for plant organ formation (Fernie and Willmitzer, 2001; Wang et al., 2012). Plant hormones are also important for plant development, and many hormones such as GA, auxin and ABA, cytokinins and jasmonic acid are involved in stolon formation (Gray, 2004). In addition, transcription factors such as the homeobox and MADS-box genes also play important roles in stolon formation (Chen et al., 2003; Abelenda et al., 2011).

*Tulipa edulis* is an important crop in TCM. Due to its low reproduction rate, the overexploitation of the wild *T. edulis* is causing the natural resources of the crop to decrease rapidly. Stolon is one of the main asexual reproductive organs of *T. edulis*. In the absence of relevant genomic data, the molecular mechanism and genetic interactions of stolon formation in *T. edulis* are not clear. In this study, three cDNA libraries of *T. edulis* stolon samples from three developmental stages were constructed for transcriptome sequencing. A total of 15.49 Gb of raw data were generated, and 74,006 unigenes were assembled in *T. edulis.* Approximately 38% of the unigenes (28,665 of 74,006) were annotated in a sequence similarity search of five public databases, and for those remaining, most of the unigenes are unique to *T. edulis.* Although most of the unigenes have not been annotated, these unigenes provide a valuable resource for the study of the genetic diversity of *T. edulis*.

To date, molecular marker techniques such as RFLP, RAPD, SNP, and SSR can be widely used in genetic diversity analysis. SSR markers have the advantage of having good repeatability, high reliability, and high polymorphism, which are all widely used for germplasm identification, genetic linkage map construction and marker-assisted breeding (Kalia et al., 2011). To the best of our knowledge, this is the first report of SSR markers in *T. edulis*. The formation of SSR repeat types showed an observable base preference for G and C content. In addition to its dominance in *T. edulis*, the CCG/CGG repeat motif is also dominant in rice and other monocotyledonous plants (Morgante et al., 2002; Song et al., 2005). The tri-, mono-, and di-nucleotide repeats contributed to the major proportion of SSRs in *T. edulis*, as the tri-nucleotide, in particular, accounted for more than half of the total SSRs. The short repeat SSR motifs also occurred with a high frequency in rice (McCouch et al., 2002) and celery (Li M.Y. et al., 2014). In contrast, the tetra-, pebta-, and hexa-nucleotide accounted for a large proportion of SSRs in rodentia (Tóth et al., 2000), arthropod (Tóth et al., 2000), and rhizobium (Gao et al., 2008). A larger number of short repeat motifs suggests that the species had a relatively higher level of evolution, while species with a large number of long repeat motifs might have a lower mutation frequency or a shorter evolutionary time (Sia et al., 1997; Harr and Schlötterer, 2000; Tóth et al., 2000; Gao et al., 2008). Therefore, we inferred that *T. edulis* maybe located at a higher level of biological evolution.

Function annotation provides reliable reference information for the understanding of gene function in stolon formation. Based on the GO categories, many DEGs participated in “metabolic process,” “cellular process,” and “response to stimulus.” COG annotation showed that the most frequent annotations of DEGs were “carbohydrate transport and metabolism” and “amino acid transport and metabolism.” Moreover, KEGG pathway analysis showed that DEGs were involved in “starch and sucrose metabolism,” “galactose metabolism,” “glycolysis/gluconeogenesis” and “plant hormone signal transduction.” In previous studies, the synthesis and metabolism of sugar and amino acid were found to be essential during plant development (Shewry, 2003). Starch and sucrose were responsible for subterranean organ formation in potato (Ferreira et al., 2010) and sweetpotato (Firon et al., 2013), and bulblet formation and development in *Lilium davidii var. unicolor* (Li X.Y. et al., 2014). These studies indicate that sucrose serves as a critical signaling molecule for cellular metabolic status (Smeekens and Hellmann, 2014). Similar findings were also reported in some bulbous plants such as *Gladiolus hybridus* (He et al., 2008) and *Tulipa gesneriana* (Yu et al., 2009). To further analyze the regulation of DEGs, we selected DEGs to verify their expression patterns. Genes related to sugar synthesis and metabolism showed significant changes during development. Some were robustly expressed in the initial stage, while some genes played regulatory roles in the later stages. As an important reproductive organ, stolon development needs a large amount of energy storage and consumption. The regulation of multiple genes is required to maintain the normal synthesis and metabolism of starch and sucrose in plant organs to ensure normal development. Naoumkina et al. (2013) found that in the *Li2* mutant, the levels of nitrogen-containing amino acids such as glutamate, glutamine, aspartate and asparagine were significantly perturbed, emphasizing the role of nitrogen metabolism and asparagine synthase in cotton fiber development. We also analyzed the expression of some genes related to amino acid metabolism and found three genes (*Te83464*, *Te91383*, *Te9579*5) that were differentially expressed but were maintained at high expression levels during development, suggesting that these genes play important roles in the regulation of the amino acid metabolism.

Hormones are necessary for plant development. Several hormones including auxin, ethylene, and JA have been reported to play important roles in bulb development (Gray, 2004). An improvement in tulip bulb formation was induced by the addition of auxin into a liquid medium (Podwyszynska, 2004). Pierik and Steegmans (1975) found that auxins promoted the efficiency of bulb formation in hyacinth, and bulbing in lily depended on the presence of exogenous auxins, mainly, naphthalene acetic acid (NAA) (Aartrijk and Blom-Barnhoorn, 1981). Currently, many hormone-responsive genes such as ARFs, Aux/IAAs, and ABR1 have been reported to participate in the specific development of plant organs (Okushima et al., 2007; Chapman and Estelle, 2009; García et al., 2014). Several genes involved in hormone signaling that were detected in this study were differentially expressed during development, which enriched our study of hormone regulation in *T. edulis*.

## Conclusion

Our data provide a comprehensive transcriptomic resource for studies of the molecular mechanisms of stolon formation in *T. edulis*. Using the Illumina platform, this work presents the first *de novo* transcriptome sequencing analysis of *T. edulis*. A total of 15.49 Gb of data were generated and assembled into 74,006 unigenes. By comparing three transcriptome libraries, we screened a large number of DEGs related to stolon formation. Functional annotation showed that the DEGs may be involved in cell growth, sugar synthesis and metabolism, amino acid transport and metabolism, and plant hormone regulation, among other processes. qRT-PCR analysis verified that the DEGs may be involved in stolon formation. The data generated by this study provides an abundant resource for further molecular and genetic studies in *T. edulis*.

## Author Contributions

QG and ZZ conceived and designed the research. ZZ and YM collected samples, generated experimental data, performed the entire data analysis, and drafted earlier versions of the manuscript. ZZ, YM, and YZ partially revised the manuscript. XY and YS were involved in the sample collection. All authors read, reviewed and approved the final manuscript. All of the authors agreed on the content of the paper and declare no conflicting interests.

## Conflict of Interest Statement

The authors declare that the research was conducted in the absence of any commercial or financial relationships that could be construed as a potential conflict of interest.
