# Supplementary material for: Transcriptome Analysis of Differentially Expressed Genes Provides Insight into Stolon Formation in Tulipa edulis
Source: Front Plant Sci. 2016 Mar 31;7:409. doi: 10.3389/fpls.2016.00409 (PMC4814499; doi:10.3389/fpls.2016.00409)
Supplement: Supplementary file 1 [file Table_1.DOCX]

**TABLE S1 Genes verified by qRT-PCR and primers used for qRT-PCR during *T. edulis* stolon formation.**

| Gene ID | Forward (5’-3’) | Reverse (5’-3’) |
| --- | --- | --- |
| *Te63130* | AATTCCATGAAGGAGGATGC | CTGGCTCCTTCAACCTAAGC |
| *Te97586* | ATTGGTGGTACAATTTCGCA | TGCTGGAGACACACAGTTCA |
| *Te97174* | ATACCCGCGATTACAGTTCC | CTTTCCTCTCCGACCTCAAG |
| *Te85890* | ATGAGCATGAGAACGACAGC | GCGATTTGTTGCTCAATCAC |
| *Te93471* | TGTTGTTCTGAGCCCATCAT | TTGGTGCTGTGCTGATCATA |
| *Te85890* | ATGAGCATGAGAACGACAGC | GCGATTTGTTGCTCAATCAC |
| *Te88793* | CTGGTGAGCGTGAAGAAGAG | TCCATCTAGGGTTGCTTCATC |
| *Te94375* | AGCTGGTGAAGGTGTCAGTG | ACCGGATACGGAGAGAACAG |
| *Te76663* | CCGGGTTGTACTGGTAGAAGA | GCCGGAGAAGAACTGGATAG |
| *Te99064* | CATCGTAGCGGAGTTTGAGA | ACCAAATGGGCCTAAGACAC |
| *Te81963* | TGCTTCCGACAATTCTCTTG | CTGAGGACTCATTTCTGCCA |
| *Te96901* | TTGGTAACTTGGATGACCGA | TTTGCCAGTTCTCTCAATCG |
| *Te80628* | TGGGCAGGGTATAAGGTGAT | TCGTACAATCACAGCCCTTC |
| *Te98020* | TCTCGCAAGCTCCAGTACAA | TGGAATTGAGCTCTTTCTCG |
| *Te98020-2* | CCTCCTTCGAGTATGTGGGT | CGCTCGAGTTATCTTCACCA |
| *Te84600* | ATGATAGGGTTTGGGAGTGG | TTCACGGTTCTCACACACCT |
| *Te89964* | TTTGGTGAGAAGCCAGACAG | ATGGCGGTCTCTACCGTAAC |
| *Te83464* | AACCTTCGTGTTAGGATGCC | TTCAATGGAGATTGGCAGAA |
| *Te91383* | CGCTCCCAGAATGGTTATTT | CTTCGCAACCAACTGAGAAA |
| *Te95795* | GGCTGCTAAGCTGGAGTTCT | GTTGTCTTTCCCGGAGTGAT |
| *actin-1* | TGTATGTTGCCATTCAGGCT | ATCACCAGAATCCAGCACAA |
