# Supplementary material for: Transcriptome Analysis of Differentially Expressed Genes Provides Insight into Stolon Formation in Tulipa edulis
Source: Front Plant Sci. 2016 Mar 31;7:409. doi: 10.3389/fpls.2016.00409 (PMC4814499; doi:10.3389/fpls.2016.00409)
Supplement: Supplementary file 2 [file Table_2.DOCX]

**TABLE S2 Gene annotation of selected DEGs during *T. edulis* stolon formation.**

| Gene ID | Predicted | Annotation |
| --- | --- | --- |
| *Te63130* | V-type proton ATPase | Plant-type cell wall biogenesis |
| *Te97586* | Momilactone A synthase | Oxidoreductase activity |
| *Te97174* | Oligopeptide transporter OPT family protein | Multidimensional cell growth; cell tip growth |
| *Te85890* | Auxin transporter-like protein 3 | Multidimensional cell growth; cell wall organization |
| *Te93471* | Wallsarethin 1 | Auxin polar transport; positive regulation of auxin metabolic process |
| *Te85890* | Auxin transporter-like protein 3 | Auxin-activated signaling pathway; auxin polar transport |
| *Te88793* | Arachidonic acid-induced DEA1 precursor | Regulation of salicylic acid metabolic process |
| *Te94375* | Probable polygalacturonase non-catalytic subunit JP650 | Auxin polar transport |
| *Te76663* | Acid beta-fructofuranosidase | Carbohydrate transport and metabolism |
| *Te99064* | Beta-fructofuranosidase 1 | Carbohydrate transport and metabolism |
| *Te81963* | Sucrose 1-fructosyltransferase | Carbohydrate transport and metabolism |
| *Te96901* | Sucrose synthase | Carbohydrate transport and metabolism |
| *Te80628* | Pectinesterase 1 | Pectin catabolic process |
| *Te98020* | Soluble starch synthase 3 | Starch biosynthetic process |
| *Te98020-2* | Soluble starch synthase 3 | Starch biosynthetic process |
| *Te84600* | 3-ketoacyl-CoA synthase 6 | Polysaccharide biosynthetic process |
| *Te89964* | Oligopeptide transporter 3 | Polysaccharide biosynthetic process |
| *Te83464* | dTDP-4-dehydrorhamnose reductase | dTDP-4-dehydrorhamnose 3,5-epimerase activity |
| *Te91383* | Probable mitochondrial-processing peptidase subunit beta | Ubiquitin-dependent protein catabolic process |
| *Te95795* | Cyanoalanine synthase | Cysteine biosynthetic process |
