# Supplementary figures and images for: Transcriptome Analysis of Differentially Expressed Genes Provides Insight into Stolon Formation in Tulipa edulis
Source: Front Plant Sci. 2016 Mar 31;7:409. doi: 10.3389/fpls.2016.00409 (PMC4814499; doi:10.3389/fpls.2016.00409)

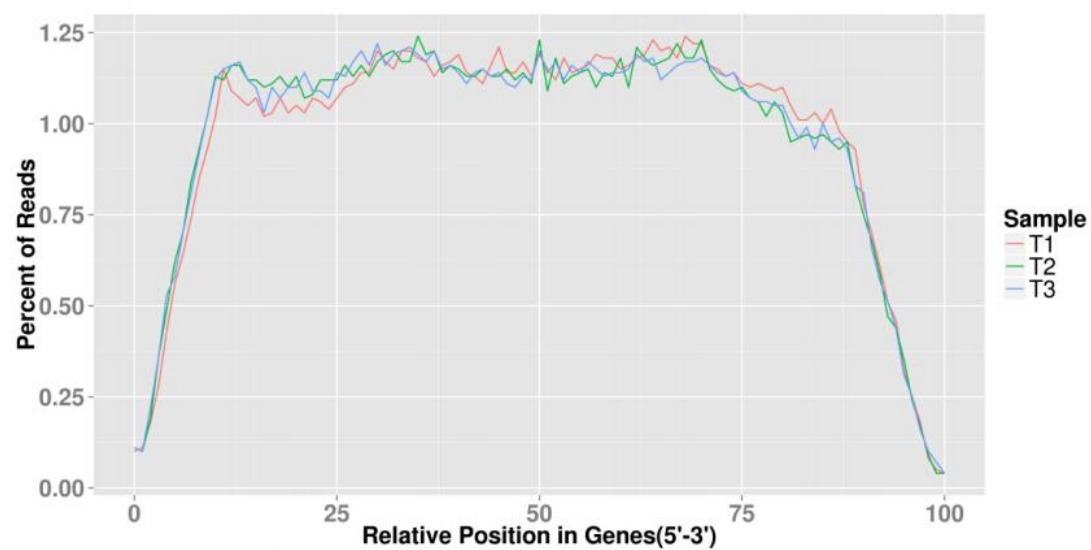

**FIGURE S1** The distribution map of randomness test of the sequence reads in *T. edulis*.

Supplement: Supplementary file 3 [file Image_1.PDF]

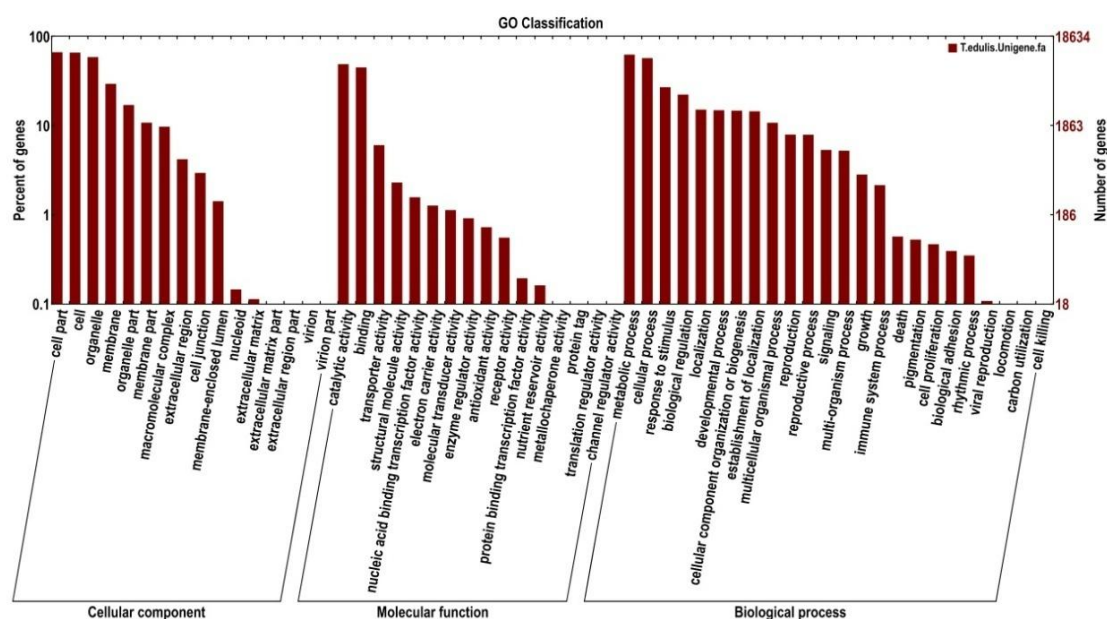

**FIGURE S2** GO classification of all unigenes in *T. edulis*.

Supplement: Supplementary file 4 [file Image_2.PDF]
