# Supplementary material for: Transcriptome Analysis of Differentially Expressed Genes Provides Insight into Stolon Formation in Tulipa edulis
Source: Front Plant Sci. 2016 Mar 31;7:409. doi: 10.3389/fpls.2016.00409 (PMC4814499; doi:10.3389/fpls.2016.00409)
Supplement: Supplementary file 5 [file Image_3.PDF]

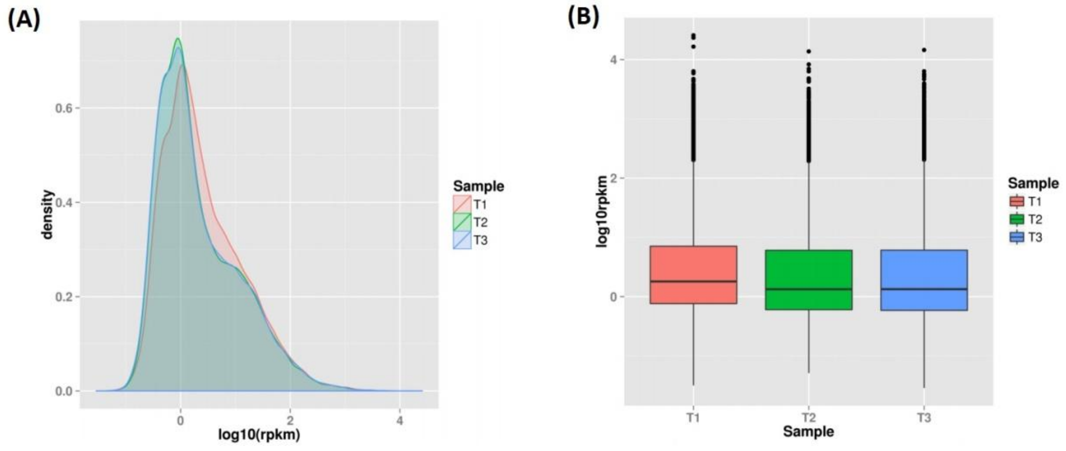

FIGURE S3 (A) RPKM density distribution of each sample of *T. edulis*. (B) RPKM box line diagram of each sample of *T. edulis*.
